# Copy number variation of *LINGO1* in familial dystonic tremor

**DOI:** 10.1212/NXG.0000000000000307

**Published:** 2019-02-04

**Authors:** Vafa Alakbarzade, Thomas Iype, Barry A. Chioza, Royana Singh, Gaurav V. Harlalka, Holly Hardy, Ajith Sreekantan-Nair, Christos Proukakis, Kathryn Peall, Lorraine N. Clark, Richard Caswell, Hana Lango Allen, Matthew Wakeling, John K. Chilton, Emma L. Baple, Elan D. Louis, Thomas T. Warner, Andrew H. Crosby

**Affiliations:** From the Medical Research (Level 4) (V.A., B.A.C., G.V.H., H.H., A.S.-N., J.K.C., E.L.B., A.H.C.), University of Exeter Medical School, RILD Wellcome Wolfson Centre, Royal Devon & Exeter NHS Foundation Trust, United Kingdom; Reta Lila Weston Institute of Neurological Studies (V.A., T.T.W.), UCL Institute of Neurology, London, United Kingdom; Department of Neurology (T.I.), Government Medical College, Thiruvananthapuram, Kerala, India; Department of Anatomy and Microbiology (R.S.), Institute of Medical Sciences, Banaras Hindu University, Varanasi, Uttar Pradesh, India; Clinical Neuroscience (C.P.), Royal Free Campus, UCL Institute of Neurology, London, United Kingdom; Institute of Psychological Medicine and Clinical Neurosciences (K.P.), Cardiff University, Cardiff, United Kingdom; Taub Institute for Research on Alzheimer's Disease and the Aging Brain (L.N.C.), Department of Pathology and Cell Biology, Columbia University Medical Center, New York, NY; Institute of Biomedical and Clinical Science (R.C., H.L.A., M.W.), University of Exeter Medical School, United Kingdom; and Departments of Neurology and Chronic Disease Epidemiology and Center for Neuroepidemiology and Clinical Neurological Research (E.D.L.), Yale School of Medicine and Yale School of Public Health, Yale University, New Haven, CT.

## Abstract

**Objective:**

To elucidate the genetic cause of a large 5 generation South Indian family with multiple individuals with predominantly an upper limb postural tremor and posturing in keeping with another form of tremor, namely, dystonic tremor.

**Methods:**

Whole-genome single nucleotide polymorphism (SNP) microarray analysis was undertaken to look for copy number variants in the affected individuals.

**Results:**

Whole-genome SNP microarray studies identified a tandem duplicated genomic segment of chromosome 15q24 present in all affected family members. Whole-genome sequencing demonstrated that it comprised a ∼550-kb tandem duplication encompassing the entire *LINGO1* gene.

**Conclusions:**

The identification of a genomic duplication as the likely molecular cause of this condition, resulting in an additional *LINGO1* gene copy in affected cases, adds further support for a causal role of this gene in tremor disorders and implicates increased expression levels of *LINGO1* as a potential pathogenic mechanism.

Tremor is a common movement disorder, and in recent years, it has become clear that essential tremor (ET) may be a group of diseases or a syndrome with clinical features that overlap with dystonia and dystonic tremor (DT).^1^ Both may be associated with isolated upper limb postural and kinetic tremor, although DT has different characteristics and is often associated with posturing or other evidence of dystonia.^2,3^ However, the considerable overlap in symptoms and signs has led to misdiagnosis of each with the other.^4^ This phenotypic heterogeneity is a complicating factor when interpreting the results of genetic studies of familial tremor. The lack of ET- or DT-specific serum, or imaging biomarkers, or defining neuropathologic features mean that clinical assessment is required to distinguish between the 2.

The *LINGO1* gene (leucine-rich repeat and Ig domain containing Nogo receptor interacting protein 1) is selectively expressed in the CNS.^5–7^ Previous studies have identified *LINGO1* as a notable genetic risk factor displaying significant association between intragenic single nucleotide polymorphism (SNP) rs9652490 and familial ET, and the same *LINGO1* SNP was replicated in independent case-control studies of ET as well as Parkinson disease.^8–13^ In the current study, we report our investigation of a large family from Southern India with multiple individuals presenting with an early-onset, bilateral, postural tremor of the upper limbs with some associated dystonic features, suggestive of DT, associated with a tandem duplication of the chromosome 15 genomic region encompassing the entire *LINGO1* gene.

## Methods

### Clinical studies

The investigated family is from Kerala, a Southern state of India, with a total of 11 affected individuals from 5 generations (figure, A) recruited with informed written consent, including permission to publish photographs. Six participants with a history of tremor (figure, A: III:5, III:9, II:9, IV:1, IV:2, and V:1) underwent a general medical and neurologic examination by the regional consultant neurologist, and a structured videotaped neurologic examination as well as Archimedes spirals were assessed by senior neurologists specializing in movement disorders (table). The videotaped neurologic examination included assessments of gait, tremor at rest, dystonia, postural tremor of the arms, and with each hand the finger-nose maneuver, the drawing of a spiral, and pouring of water. The other 5 individuals with tremor (II:1, II:6, II:10, III:16, and IV:8) had their affected status confirmed with an examination conducted by the local consultant neurologist. Four other family members (II:4, II:11, III:11, and IV:5) were examined by the same consultant neurologist and confirmed to have no evidence of tremor or dystonia.

**Figure:**
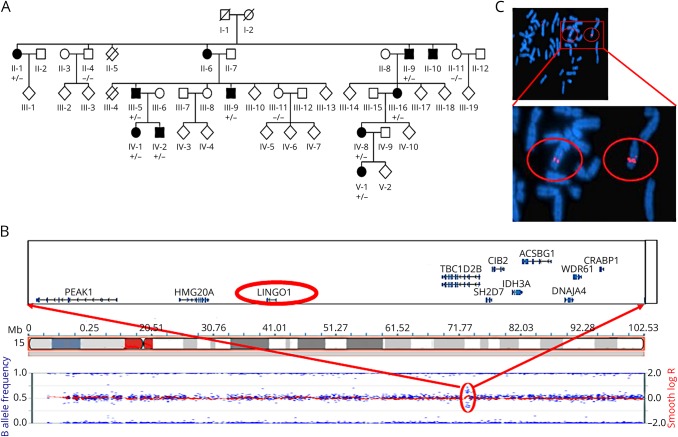
Pedigree and genomic analysis of the Indian family (A) Pedigree of large multigenerational Indian family exhibiting genotype of the affected and unaffected individuals studied using whole-genome SNP microarray analysis and FISH (“+” identifies the presence of duplication and “-” identifies wild-type allele). (B) Chromosome 15q24.3-q25.1 duplicated region highlighted with red circle encompassing *LINGO1* gene. (C) FISH confirming presence of the duplication on chromosome 15 in affected individuals showing enhanced signal for the derivative chromosome (circled to right of figure). FISH = fluorescent in situ hybridization.

**Table:**
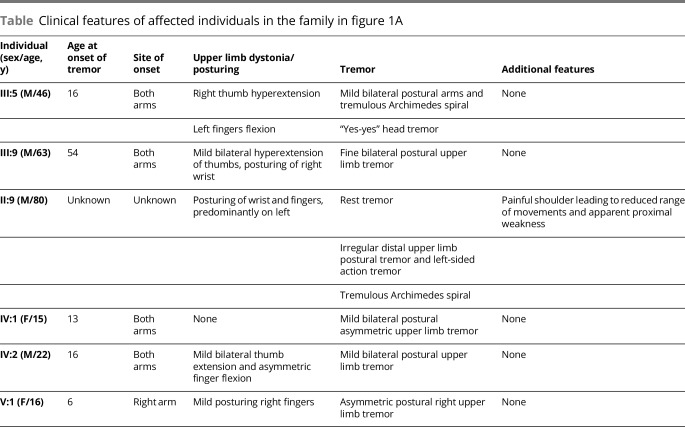
Clinical features of affected individuals in the family in figure 1A

### Microarray analysis and fluorescent in situ hybridization

Venous blood was collected in EDTA and PAXgene Blood RNA tubes (PreAnalytiX), and skin biopsy was performed in 3 cases (III:5, IV:2, and II:9) for fluorescent in situ hybridization (FISH) studies. Genomic DNA and RNA samples were extracted from peripheral blood following standard protocols. Genome-wide SNP genotyping was undertaken using Illumina HumanCytoSNP-12 v2.1 SNP microarrays, and image data were processed using Illumina GenomeStudio software to generate genotype calls, B allele frequency, and logR ratio values. These were further analyzed for copy number variant (CNVs) using Illumina's KaryoStudio software. To minimize false-positive CNV calls, filtering approaches were applied to exclude smaller repeats (<100 kb) and common CNVs from Database of Genomic Variants (dgv.tcag.ca/dgv/app/home). For FISH analysis, BlueFISH probe RP11-114H24 was used to confirm chromosomal duplication in individuals II:9, III:5, and IV:2.

### Genomic library preparation

Genomic DNA (∼3 μg) was fragmented by sonication using a Bioruptor (UCD-200; Diagenode, Seraing, Liege, Belgium) to an average size of ∼400 base pairs (bp), and DNA was purified using 1.2 volumes Ampure XP (Agencourt). End repair and dA tailing were carried out using NEBNext modules (New England Biolabs, Hertfordshire, United Kingdom), with DNA purification using 1.8 volumes Ampure after each step. DNA fragments were then ligated to paired-end adapters for Illumina sequencing using Epicentre Fast-Link DNA ligation kit (Cambio, Cambridgeshire, United Kingdom). The entire ligation reaction was separated on a 1.2% agarose gel, and DNA fragments in the size range ∼400–450 bp were excised. DNA was extracted from the gel slice using the QIAquick gel extraction kit (Qiagen, Manchester, United Kingdom) and analyzed on a high-sensitivity chip for the Agilent Bioanalyzer 2100 (Agilent Technologies, Santa Clara, CA). Adapter-ligated DNA (50 ng) was amplified for 6 cycles using Herculase II Fusion DNA Polymerase (Agilent Technologies) and Illumina PE_PCR primers 1 and 2, then diluted for sequencing. Sequencing was carried out by the Exeter Sequencing Service (School of Biosciences, University of Exeter, Exeter, United Kingdom) on an Illumina HiSeq2500 using 100-bp paired-end reads in rapid run mode, yielding a total of 23.8 gigabases (Gb) of sequence. Reads were aligned to the human reference genome (build GRCh37/hg19) using the BWA alignment tool, and duplicate reads were removed using Picard, yielding an average coverage depth of ∼7.5X reads per base.

### Standard protocol approvals, registrations, and patient consents

The study was approved and performed under the ethical guidelines issued by our institutions for clinical studies, with written informed consent obtained from all participants for genetic studies.

## Results

### Clinical studies

The extended pedigree of the family is presented in figure A, and clinical details of the tremor and salient neurologic features are presented in table, including age at onset. The clinical examination was supplemented by review of videotaped neurologic examinations of individuals III:5, III:9, IV:1, IV:2, and V:1 by 2 neurologists with specialization in movement disorders (E.D.L., T.T.W.). The presence of sustained postures of the hands/wrists in 4 of 5 affected individuals as well as a yes-yes head tremor in one additional individual with abnormal hand postures are atypical for ET and confirmed the diagnosis of DT. Two exemplar videos of individuals III:5 and II:9 demonstrate the dystonic posturing (video 1). On examination of II:1, II:6, II:9, II:10, III:16, and IV:8, mild bilateral postural hand limb tremor, with variable degree of thumb or index finger posturing, was detected. Individuals II:4, II:11, III:11, and IV:5 displayed no tremor or posturing. Five of the 6 affected individuals had postural tremor in association with dystonic posturing and only one had isolated postural tremor (table). Although EMG can be useful in differentiating ET from DT, the obvious posturing in the upper limb in all cases obviate the need for this additional test. None of the affected individuals had other abnormal salient neurology, including evidence of parkinsonism or abnormal eye movements.

10.1212/000307_Video_1 Video 1 Download Supplementary Video 1 via http://dx.doi.org/10.1212/000307_Video_1

### Genetic studies

Genome-wide SNP microarray analysis (Illumina Human CytoSNP-12v2.1) of all available family members identified a single notable genomic rearrangement in affected family members, a duplication of chromosome 15q24.3-q25.1 in all 9 affected family members (figure, A and B, figure e-1A, links.lww.com/NXG/A139). This duplicated region, delimited by KaryoStudio, was found to contain 14 RefSeq genes (figure, B; table e-1, links.lww.com/NXG/A139) and was confirmed in affected family members using FISH analysis (using BlueFish probe RP11-114H24; chr15:78146252-78322027, figure, C). Targeted next-generation sequencing of an affected patient (III:16) was then used to precisely map the chromosomal breakpoints of the duplication. This identified read pairs mapping ∼550 kb apart in reverse-forward rather than forward-reverse orientation, indicative of a tandem duplication event (figure, B; figure e-1A, links.lww.com/NXG/A139). The exact coordinates of the rearrangement event (chr15:77775483-78331797dup [hg19]) were identified in reads spanning each breakpoint (figure e-1B, links.lww.com/NXG/A139), located in genes *HMG20A* and *TBC1D2B*, respectively. The read count across the region indicates an average coverage increase from approximately 7.5X to 10X, broadly consistent with an expected ∼50% increase in the number of reads for a heterozygous duplication. The 14 genes located within the duplicated region (figure, B) were investigated for candidacy, which identified only a single stand-out candidate with a role in brain development or function (*LINGO1*).

PCR primers were positioned to produce an amplicon specific to the genetic sequence created at the boundary between the tandem duplications, generating a product of 1,184 bp arising from this de novo event. Dideoxy sequencing of this PCR product confirmed the location of the duplication event to chr15:77775487-78331797 [hg19]. This facilitated the genotyping of family members using a simple PCR-based strategy to identify family members who have inherited the rearragnement. PCR analysis on all individuals from the pedigree confirmed co-segregation of the rearrangement in affected family members (figure, A), as well as its absence from 100 age-matching healthy controls from the same geographical region.

## Discussion

Here, we investigated an extended Indian family with multiple individuals affected by a movement disorder involving tremor. The presence of abnormal upper limb postures in all 11 affected individuals along with the presence of only mild tremor is most consistent with DT rather than ET in this family.^1^

As noted above, previous genome-wide association studies have demonstrated association between DNA sequence variants in the *LINGO1* gene and ET. With the case we report now, the potential involvement of a duplication involving the *LINGO1* gene may suggest a similar genetic and molecular mechanistic basis to some cases of ET and DT. LINGO1 protein is known to interact with Nogo-66 receptor (NgR1) and p75 neurotrophin receptor (p75^NTR^) or TROY, to form an NgR1 complex, which binds to inhibitory molecules such as Nogo-A.^14,15^ The NgR1 complex Nogo-A activates RhoA as a negative regulator for neuronal survival, axonal regeneration, oligodendrocyte maturation, and neuronal myelination.^15–21^ Notably, the p75NTR-sortilin (*SORT1*) receptor complex has previously been implicated in tremor phenotypes via a p.Gly171Ala SORT1 missense variant, which impaired expression of both protein members of the sortilin-p75^NTR^ complex.^22^ As such, *LINGO1* represents a strong candidate gene for involvement in movement disorders such as DT and ET, and consistent with this, previous genome-wide association studies have indeed indicated an association between variants in *LINGO1* and ET.^9–13^ However, all identified risk variants are located in *LINGO1* intronic regions, and subsequent sequencing of *LINGO1* coding exons in ET patients have failed to identify putative pathogenic sequence variants.^9–11,23,24^

The studies reported here define a previously undescribed duplication event encompassing the *LINGO1* gene present in multiple individuals of this extended Indian family. While it is not possible to exclude involvement of other genes in the duplicated region, LINGO1 represents the only stand-out functional candidate in the region. This finding lends us to speculate that increased transcription and ensuing gene activity deriving from the additional (trisomic) copy of *LINGO1* are the likely pathogenic cause of this condition. This indicates that the previously identified intronic *LINGO1* gene variants displaying association with ET may result in the condition by directly influencing (or being in linkage disequlibrium with variants that directly influence) native gene transcription. Consistent with this notion, a previous study detected increased levels of LINGO1 in the cerebellum ET patients.^25^ Thus, while the outcome of duplication of the other genes located within the genomic region defined in our study requires further exploration, our data, combined with existing studies of *LINGO1* in ET, indicate that hypermorphic mutation leading to increased transcriptional or protein activity of LINGO1 represents a likely pathogenic cause. This may manifest itself via an increased density of basket cell processes generated by an increased LINGO1 dosage effect, leading to an inhibitory effect on Purkinje cell GABAergic neurones. Decreased or inhibited cerebellar inhibitory output has been demonstrated to cause postural, kinetic tremor as well as motor incoordination in GABAA α1 knockout mice.^24^ In the olivary animal models of action tremor, it has also been shown that such action tremor is a primarily electrophysiologic entity caused by abnormal olivary-cerebellar excitatory output.^26^ Thus, it is tempting to speculate that this may result from an imbalance of excitatory-inhibitory interneuronal connection secondary to the dosage effect of LINGO1, and it would be of interest to observe the effect of LINGO1 protein agonists on the olivary animal models of ET to investigate this hypothesis. The data from this study are consistent with this notion and demonstrate *LINGO1* copy number gain in familial postural tremor, suggestive of a LINGO1 dosage disease mechanism.

## Glossary

CNV: copy number variant
DT: dystonic tremor
ET: essential tremor
FISH: fluorescent in situ hybridization
NgR1: Nogo-66 receptor

## References

[1] Gövert F, Deuschl G Tremor entities and their classification: an update. Curr Opin Neurol 2015;28:393–399. 26110800 10.1097/WCO.0000000000000211

[2] Albanese A, Bhatia K, Bressman SB, et al Phenomenology and classification of dystonia: a consensus update. Mov Disord 2013;28:863–873. 23649720 10.1002/mds.25475 PMC3729880

[3] Stamelou M, Edwards MJ, Bhatia KP Late onset rest-tremor in DYT1 dystonia. Parkinsonism Relat Disord 2013;19:136–137. 22721973 10.1016/j.parkreldis.2012.05.026

[4] Quinn NP, Schneider SA, Schwingenschuh P, Bhatia KP Tremor: some controversial aspects. Mov Disord 2011;26:18–23. 21322015 10.1002/mds.23289

[5] Barrette B, Vallières N, Dubé M, Lacroix S Expression profile of receptors for myelin-associated inhibitors of axonal regeneration in the intact and injured mouse central nervous system. Mol Cell Neurosci 2007;34:519–538. 17234430 10.1016/j.mcn.2006.12.004

[6] Inoue H, Lin L, Lee X, et al Inhibition of the leucine-rich repeat protein LINGO-1 enhances survival, structure, and function of dopaminergic neurons in Parkinson's disease models. Proc Natl Acad Sci USA 2007;104:14430–14435. 17726113 10.1073/pnas.0700901104 PMC1955463

[7] Llorens F, Gil V, Iraola S, et al Developmental analysis of Lingo-1/Lern1 protein expression in the mouse brain: interaction of its intracellular domain with Myt1l. Dev Neurobiol 2008;68:521–541. 18186492 10.1002/dneu.20607

[8] Louis ED, Faust PL, Vonsattel JP, et al Neuropathological changes in essential tremor: 33 cases compared with 21 controls. Brain 2007;130:3297–3307. 18025031 10.1093/brain/awm266

[9] Stefansson H, Steinberg S, Petursson H, et al Variant in the sequence of the LINGO1 gene confers risk of essential tremor. Nat Genet 2009;41:277–279. 19182806 10.1038/ng.299 PMC3740956

[10] Tan EK, Teo YY, Prakash KM, et al LINGO1 variant increases risk of familial essential tremor. Neurology 2009;73:1161–1162. 19805735 10.1212/WNL.0b013e3181bacfc9 PMC2890998

[11] Vilarino-Guell C, Wider C, Ross OA, et al LINGO1 and LINGO2 variants are associated with essential tremor and Parkinson disease. Neurogenetics 2010;11:401–408. 20369371 10.1007/s10048-010-0241-x PMC3930084

[12] Vilarino-Guell C, Ross OA, Wider C, et al LINGO1 rs9652490 is associated with essential tremor and Parkinson disease. Parkinsonism Relat Disord 2010;16:109–111. 19720553 10.1016/j.parkreldis.2009.08.006 PMC2844122

[13] Radovica I, Inashkina I, Smeltere L, Vitols E, Jankevics E Screening of 10 SNPs of LINGO1 gene in patients with essential tremor in the Latvian population. Parkinsonism Relat Disord 2012;18:93–95. 21741293 10.1016/j.parkreldis.2011.06.006

[14] Shao Z, Browning JL, Lee X, et al TAJ/TROY, an orphan TNF receptor family member, binds Nogo-66 receptor 1 and regulates axonal regeneration. Neuron 2005;45:353–359. 15694322 10.1016/j.neuron.2004.12.050

[15] Mi S, Sandrock A, Miller RH LINGO-1 and its role in CNS repair. Int J Biochem Cell Biol 2008;40:1971–1978. 18468478 10.1016/j.biocel.2008.03.018

[16] McGee AW, Strittmatter SM The Nogo-66 receptor: focusing myelin inhibition of axon regeneration. Trends Neurosci 2003;26:193–198. 12689770 10.1016/S0166-2236(03)00062-6

[17] Yiu G, He Z Glial inhibition of CNS axon regeneration. Nat Rev Neurosci 2006;7:617–627. 16858390 10.1038/nrn1956 PMC2693386

[18] Lee X, Yang Z, Shao Z, et al NGF regulates the expression of axonal LINGO-1 to inhibit oligodendrocyte differentiation and myelination. J Neurosci 2007;27:220–225. 17202489 10.1523/JNEUROSCI.4175-06.2007 PMC6672289

[19] Llorens F, Gil V, del Río JA Emerging functions of myelin-associated proteins during development, neuronal plasticity, and neurodegeneration. FASEB J 2011;25:463–475. 21059749 10.1096/fj.10-162792

[20] Jepson S, Vought B, Gross CH, et al LINGO-1, a transmembrane signaling protein, inhibits oligodendrocyte differentiation and myelination through intercellular self-interactions. J Biol Chem 2012;287:22184–22195. 22514275 10.1074/jbc.M112.366179 PMC3381180

[21] Lööv C, Fernqvist M, Walmsley A, Marklund N, Erlandsson A Neutralization of LINGO-1 during in vitro differentiation of neural stem cells results in proliferation of immature neurons. PLoS One 2012;7:e29771. 22235341 10.1371/journal.pone.0029771 PMC3250485

[22] Sanchez E, Bergareche A, Krebs CE, et al SORT1 mutation resulting in sortilin deficiency and p75(NTR) upregulation in a family with essential tremor. ASN Neuro 2015;7:1759091415598290. 26297037 10.1177/1759091415598290 PMC4550298

[23] Clark LN, Park N, Kisselev S, Rios E, Lee JH, Louis ED Replication of the LINGO1 gene association with essential tremor in a North American population. Eur J Hum Genet 2010;18:838–843. 20372186 10.1038/ejhg.2010.27 PMC2987362

[24] Thier S, Lorenz D, Nothnagel M, et al LINGO1 polymorphisms are associated with essential tremor in Europeans. Mov Disord 2010;25:717–723. 20310002 10.1002/mds.22887

[25] Delay C, Tremblay C, Brochu E, et al Increased LINGO1 in the cerebellum of essential tremor patients. Mov Disord 2014;29:1637–1647. 24531928 10.1002/mds.25819

[26] Wang GJ, Yang P, Xie HG Gene variants in noncoding regions and their possible consequences. Pharmacogenomics 2006;7:203–209. 16515399 10.2217/14622416.7.2.203

